# Supplementary material for: Stromal androgen signaling acts as tumor niches to drive prostatic basal epithelial progenitor-initiated oncogenesis
Source: Nat Commun. 2022 Nov 2;13:6552. doi: 10.1038/s41467-022-34282-w (PMC9630272; doi:10.1038/s41467-022-34282-w)
Supplement: Supplementary file 1 — Supplementary Information [file 41467_2022_34282_MOESM1_ESM.pdf]

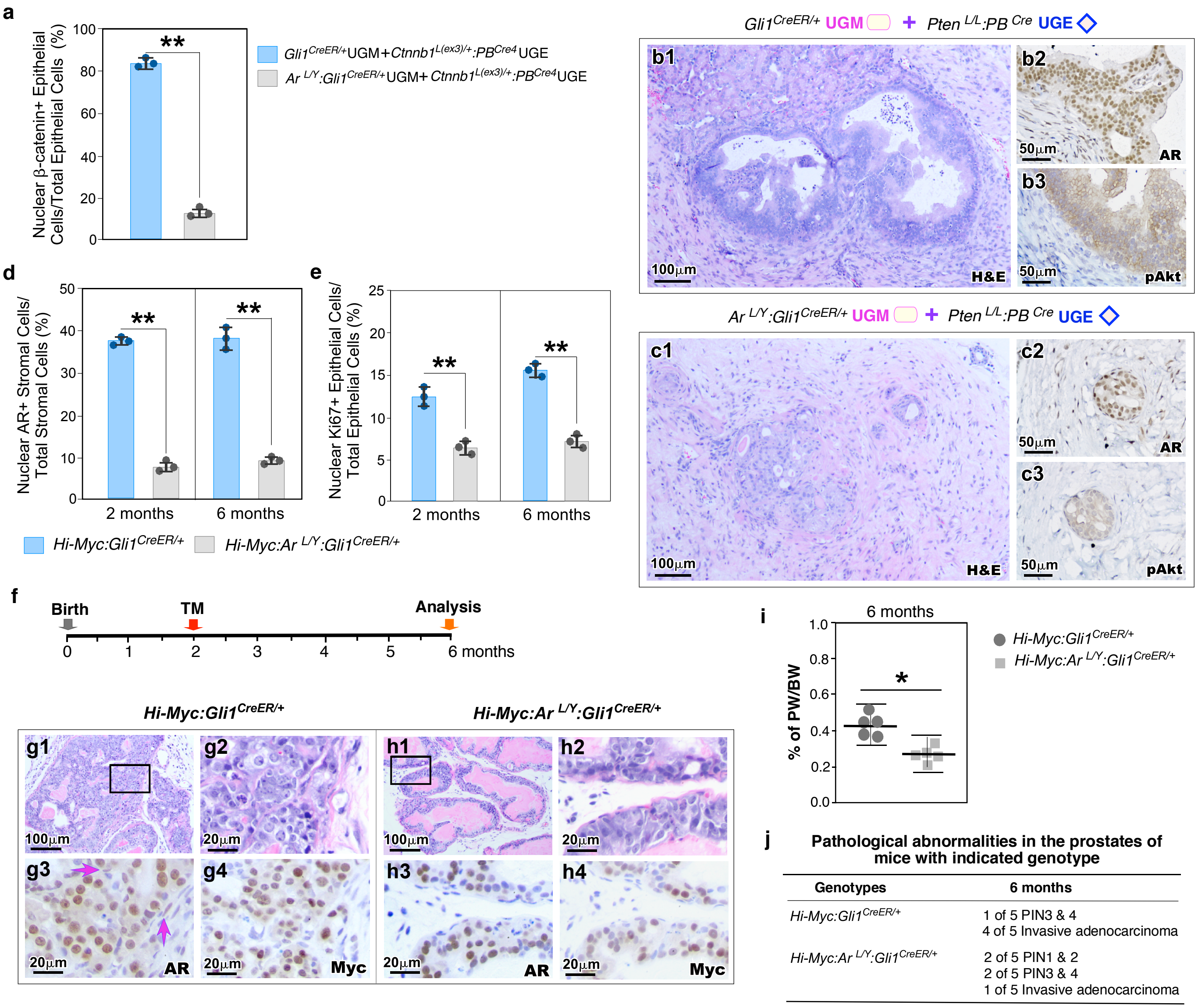

Supplementary Fig.1 Deletion of androgen receptor (AR) expression in Gli1-lineage cells impairs prostatic tumorigenesis and development. a Quantification of the percentage of nuclear β-catenin positive epithelial cells per total epithelial cells from *Ctnnb1*$^{L(ex3)/+}$*:PB*^{Cre4} urogenital sinus epithelium (UGE) combined with urogenital sinus mesenchyme (UGM) from *Gli1*$^{CreER/+}$ or *Ar*$^{L/Y}$*:Gli1*$^{CreER/+}$ embryos after activation of *Gli1*$^{CreER/+}$. Data are represented as mean ± SD of three biological replicates. Two-sided Student's *t*-test, $^{**}p$ < 0.01. **b-c** Representative hematoxylin-eosin (H&E) and immunohistochemical (IHC) analyses for AR and phospho-Akt (pAkt) on xenograft recombinants from *Pten*$^{L/L}$*:PB*^{Cre4} UGE + *Gli1*$^{CreER/+}$ UGM or *Ar*$^{L/Y}$*:Gli1*$^{CreER/+}$ UGM combinations. Scale bars, 100 µm and 50 µm. **d-e** Quantification of the percentage of nuclear AR^{+} stromal cells per total stromal cells or nuclear Ki67^{+} epithelial cells per total epithelial cells in the prostates of 2- and 6-month-old *Hi-Myc:Gli1*$^{CreER/+}$ and *Hi-Myc:Ar*$^{L/Y}$*:Gli1*$^{CreER/+}$ mice after activation of *Gli1*$^{CreER/+}$ at 2 weeks of age. Data are represented as mean ± SD of three biological replicates, and using two-sided Student's *t*-test, $^{**}p$ < 0.01. **f** Schematic of experimental timeline for adult *Gli1*$^{CreER/+}$ activation in *Hi-Myc:Gli1*$^{CreER/+}$ and *Hi-Myc:Ar*$^{L/Y}$*:Gli1*$^{CreER/+}$ mice. **g-h** H&E and IHC analyses for AR and Myc on adjacent prostate tissue sections of 6-month-old *Hi-Myc:Gli1*$^{CreER/+}$ and *Hi-Myc:Ar*$^{L/Y}$*:Gli1*$^{CreER/+}$ mice with *Gli1*$^{CreER/+}$ activation at 2 months of age. Purple arrows indicate stromal AR^{+} cells in *Hi-Myc:Gli1*$^{CreER/+}$ mice. Scale bars, 100 µm and 20 µm. **i** Ratio of prostate weight (PW) versus whole body weight (BW) as percentages of 6-month-old mice, which were given tamoxifen (TM) at 2 months of age. Data are represented as mean ± SD of five biological replicates, and using two-sided Student's *t*-test, $^{*}p$ < 0.05. **j** Table summarizing the pathological abnormalities in the prostates of 6-month-old *Hi-Myc:Gli1*$^{CreER/+}$ and *Hi-Myc:Ar*$^{L/Y}$*:Gli1*$^{CreER/+}$ mice after TM administration at 2 months of age. PIN, prostatic intraepithelial neoplasia. For **a**, **d**, **e**, and **i**, source data and the exact *p*-values are provided in the Source Data file.

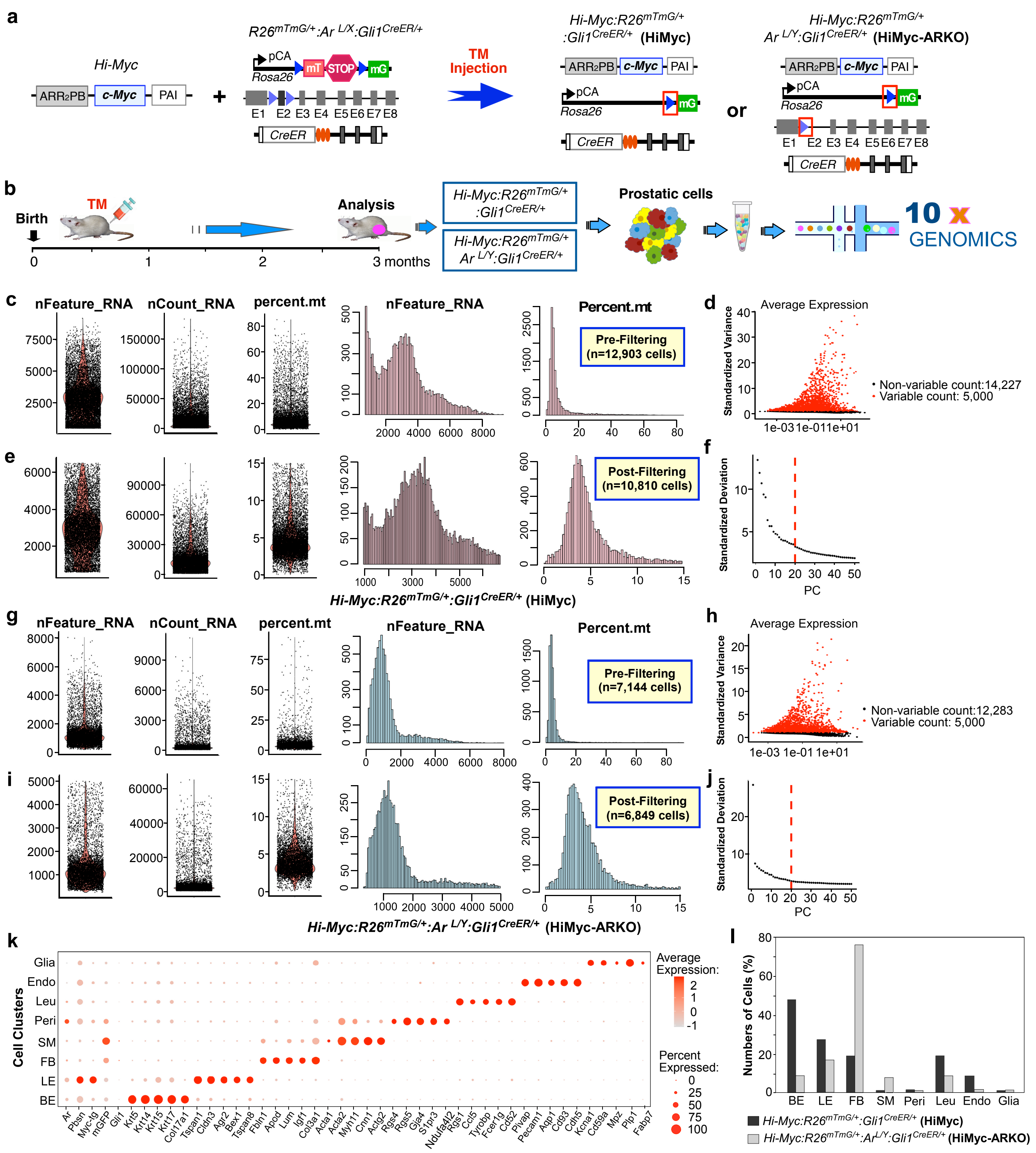

Supplementary Fig.2 Single cell RNA sequencing analyses of prostate tissues from *Hi-Myc:R26$^{mTmG/+}$:Gli1$^{CreER/+}$* and *Hi-Myc:R26$^{mTmG/+}$:Ar$^{L/Y}$:Gli1$^{CreER/+}$* mice. a Schematic of mating strategy for generating *Hi-Myc:R26$^{mTmG/+}$:Gli1$^{CreER/+}$* and *Hi-Myc:R26$^{mTmG/+}$:Ar$^{L/Y}$:Gli1$^{CreER/+}$* mice. **b** Schematic of experimental design for the single cell RNA-sequencing (scRNA-seq) analyses. **c-j** Quality controls for scRNA-seq data from *Hi-Myc:R26$^{mTmG/+}$:Gli1$^{CreER/+}$* (HiMyc) and *Hi-Myc:R26$^{mTmG/+}$:Ar$^{L/Y}$:Gli1$^{CreER/+}$* (HiMyc-ARKO) prostates. Violin and histogram plots corresponding to the number of features, counts, and the percentage of mitochondrial RNA in each cell from HiMyc prostates before filtering (**c**) and after filtering for 1,000 < nFeature < 7,000 and percent.mt < 15 (**e**). Plot of gene variability and selection of the top 5,000 variable genes from HiMyc prostates (**d**). PCA elbow plot identifying a cutoff of 20 dimensions (dims) used for further analyses of HiMyc prostates (**f**). Violin and histogram plots corresponding to the number of features, counts, and the percentage of mitochondrial RNA in each cell from HiMyc-ARKO prostates before filtering (**g**) and after filtering for 500 < nFeature < 5,000 and percent.mt < 15 (**i**). Plot of gene variability and selection of the top 5,000 variable genes from HiMyc-ARKO prostates (**h**). PCA elbow plot identifying a cutoff of 20 dims used for further analyses of HiMyc-ARKO prostates (**j**). **k** Dot plot displaying five highly specific genes in each cell type from integrated scRNAseq data of HiMyc and HiMyc-ARKO Mice. **l** Distribution of total cells for each cell type in each indicated genotypes. BE, Basal epithelial cells; LE, Luminal epithelial cells; FB, Fibroblasts; SM, Smooth muscle cells; Peri, Pericytes; Leu, Leukocytes; Endo, Vesicular endothelial cells; Glia, Glial cells.

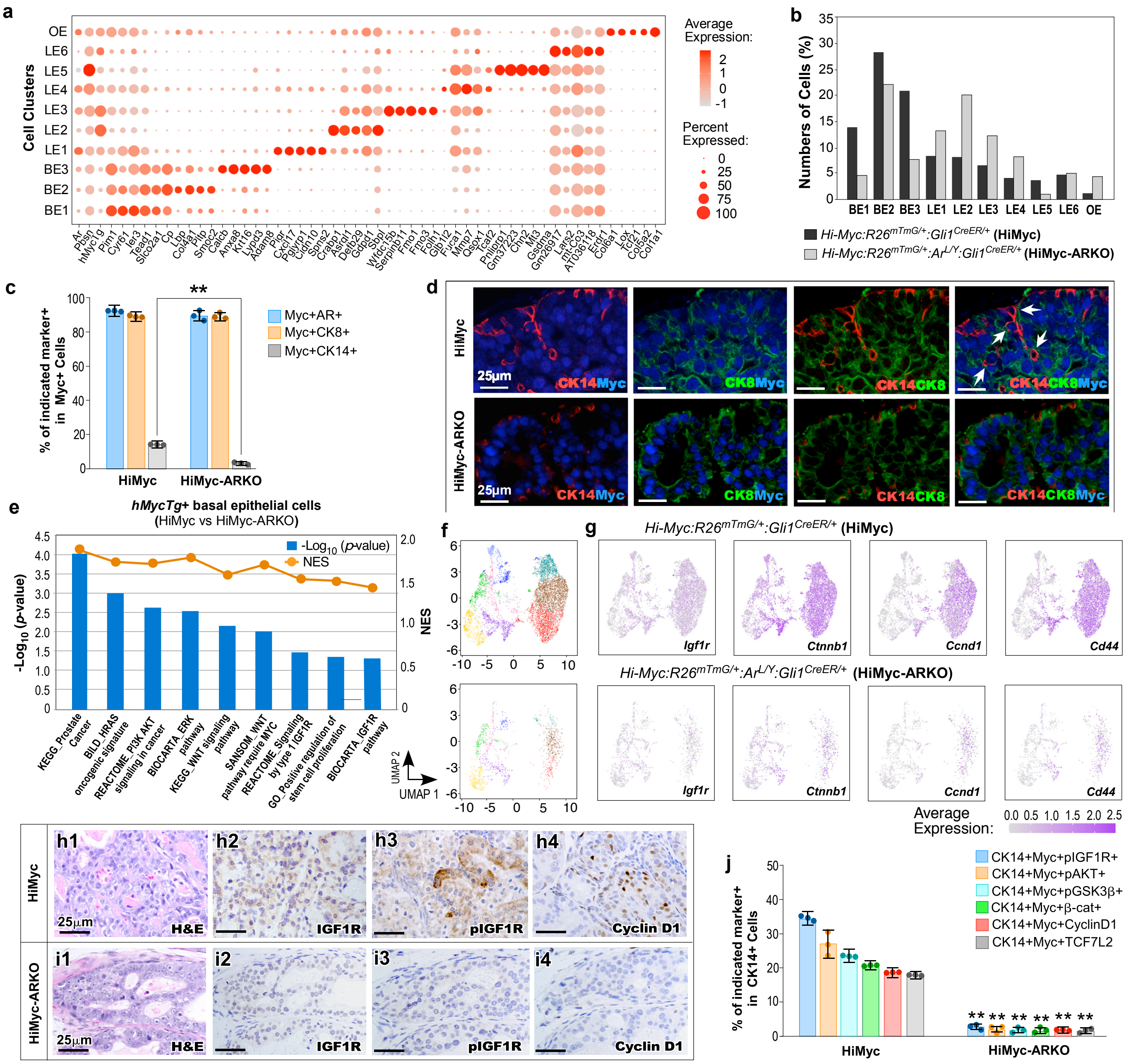

k

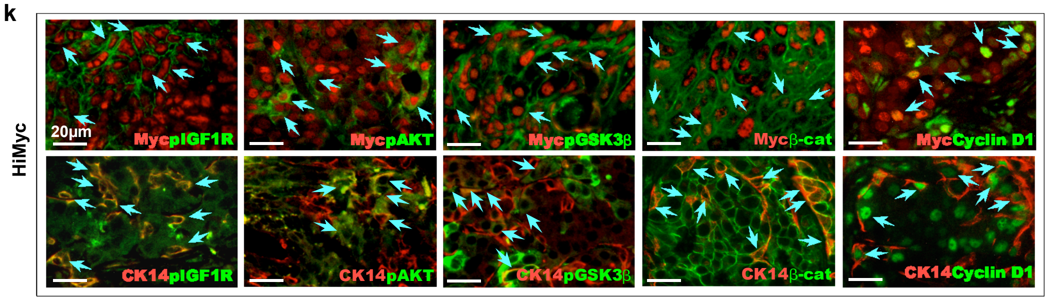

Supplementary Fig.3 Analyses of cellular properties and molecular changes of prostatic epithelial cells. a Dot plot depicting five highly specific genes in each subtype from epithelial cells in *Hi-Myc:R26* $^{mTmG/+}$*:Gli1*$^{CreER/+}$ (HiMyc) and *Hi-Myc:R26*$^{mTmG/+}$*: Ar*$^{L/Y}$*:Gli1* $^{CreER/+}$ (HiMyc-ARKO) mice. **b** Distribution of epithelial cells for each epithelial subtype in indicated genotype. **c** Quantification (**Fig. 3h**) of of Myc^{+}AR^{+}, Myc^{+}CK8^{+}, and Myc^{+}CK14^{+} double positive cells per total Myc^{+} cells in adjacent prostate tissue sections from HiMyc and HiMyc-ARKO mice. **d** Representative triple-IF staining for the indicated antibodies in adjacent prostate tissue sections from HiMyc and HiMyc-ARKO (n=3 per genotype). White arrows show triple positive cells for CK14, CK8 and Myc. Scale bars, 25µm. **e** GSEA using *p*-value based pre-ranked gene list from *hMycTg*$^{+}$ basal cells in HiMyc versus those cells in HiMyc-ARKO. Please also see Methods. **f** UMAP plots separated by indicated genotypes from epithelial cells (**Fig. 3d**). **g** Gene expression levels of *Igf1r, Ctnnb1, Ccnd1,* and *Cd44* in epithelial cells, split by indicated genotypes. **h-i** Representative images of H&E and IHC analyses for cellular markers in the figure on adjacent prostate tissue sections from indicated genotypes. Scale bars, 25µm. **j** Quantification (**Fig. 4e**) of Myc^{+}CK14^{+}pIGF1R^{+}, Myc^{+}CK14^{+}pAKT^{+}, Myc^{+}CK14^{+}pGSK3β^{+}, Myc^{+}CK14^{+}β-cat^{+}, Myc^{+}CK14^{+}Cyclin D1^{+}, and Myc^{+}CK14^{+}TCF7L2^{+} triple positive cells per total CK14^{+} cells in adjacent prostate tissue sections from different genotype mice. **k** Representative Co-IF staining for the indicated antibodies in adjacent prostate tissue sections from HiMyc and HiMyc-ARKO (n = 3 per genotype). Blue arrows show double-positive cells for the indicated antibody combinations. Scale bar, 20 µm. In **c** and **j**, data are represented as mean ± SD of three biological replicates. Two-sided Student' s *t*-test for HiMyc versus HiMyc-ARKO, $^{**}p$ < 0.01. Source data and the exact *p*-values are provided in the Source Data file.

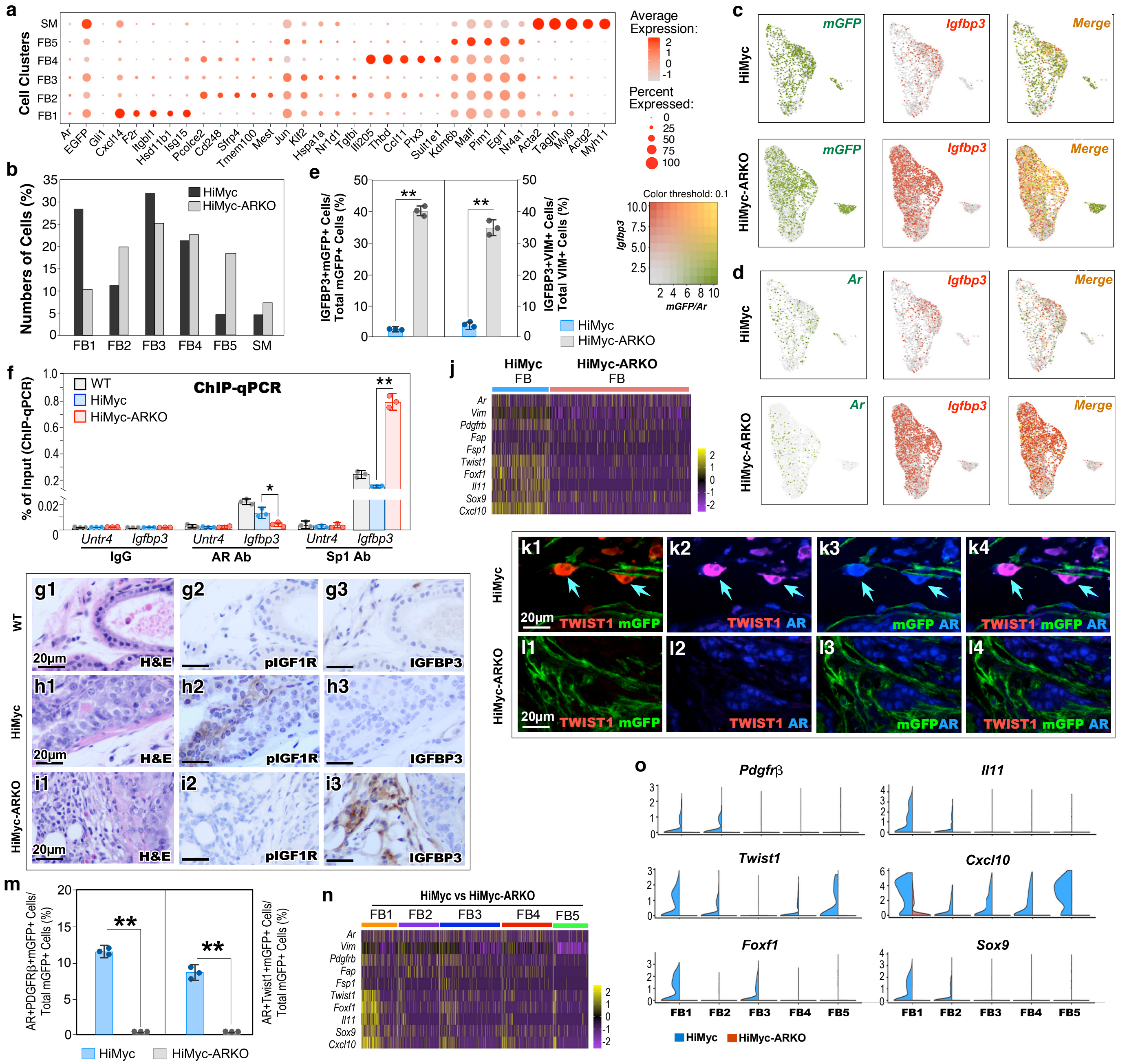

Supplementary Fig.4 Analyses of cellular properties and molecular changes of prostatic stromal cells. a Dot plot depicting five highly specific genes in each sub-cluster from fibroblast (FB) and smooth muscle (SM) cells in both *Hi-Myc:R26*$^{mTmG/+}$*:Gli1*$^{CreER/+}$ (HiMyc) and *Hi-Myc:R26*$^{mTmG/+}$*:Ar*$^{L/Y}$*:Gli1*$^{CreER/+}$ (HiMyc-ARKO) mice. **b** Distribution of FB and SM cells for each subtype from indicated genotypes. **c-d** Blended expression UMAP plots of *Igfbp3* and *mGFP* (**c**) or *Ar* (**d**) in FB and SM cells from indicated genotypes. **e** Quantification of the percentage of double positive IGFBP3^{+}mGFP^{+} per total mGFP^{+} or IGFBP3^{+}VIM^{+} per total VIM^{+} cells in the indicated genotypes. **f** AR and Sp1 Chromatin immunoprecipitation qPCR analyses of the *Igfbp3* promoter regions, and negative control (*Untr4*) shown as percent inputs. **g-i** Representative images from H&E and IHC analyses for pIGF1R and IGFBP3 using prostate tissues from 6-month-old HiMyc and HiMyc-ARKO mice with TM administration at 2 months of age. Scale bars, 20 μm. **j** Heatmap displaying gene expression as indicated above between prostatic FB cells of HiMyc and HiMyc-ARKO mice. **k-l** Representative images of triple-IF staining for TWIST1, mGFP, and AR on prostate tissues from indicated genotypes. Blue arrows indicate cells showing double- or triple-positive with the indicated genes listed. Scale bars, 20 μm. **m** Quantification for percentage of triple positive staining of AR^{+}PDGFRβ^{+}mGFP^{+} (left panel) or AR^{+}TWIST1^{+}mGFP^{+} (right panel) per total mGFP^{+} cells **n** Heatmap displaying gene expression of *Ar*, *Vim*, and CAF markers across five subtypes of total FB cells between HiMyc and HiMyc-ARKO. **o** Split-violin plots visualizing the expression levels of CAF markers in FB subtypes between two different genotypes as indicated in the figure. In **e**, **f**, and **m**, data are represented as mean ± SD of three biological replicates. Two-sided Student's t-test, $**p < 0.01$. Source data and the exact *p*-values are provided in the Source Data file.

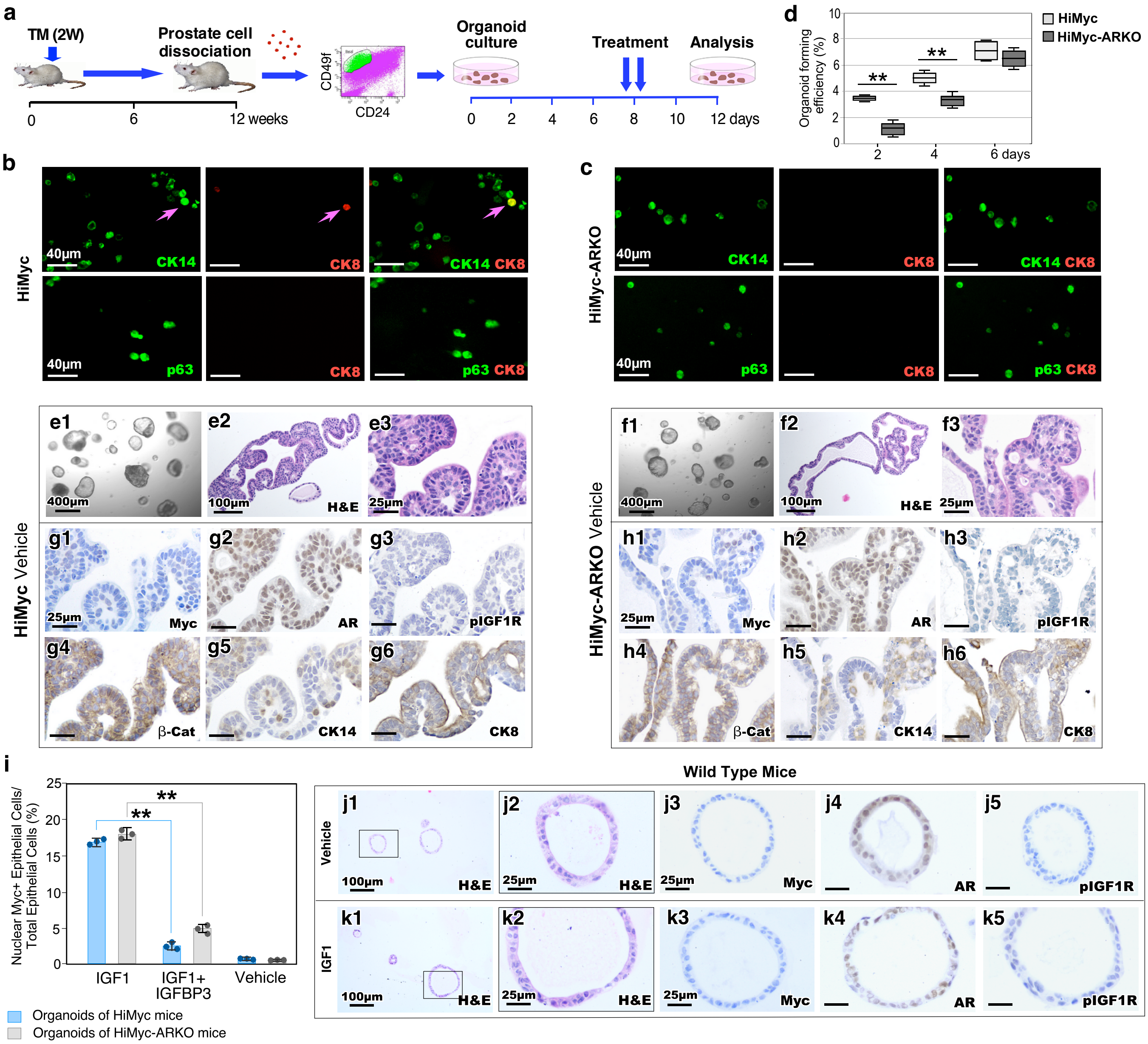

Supplementary Fig.5 Analyses of IGF signaling on oncogenic prostatic epithelial organoid growth. a Schematic of experimental design and timeline for organoid culture using prostatic CD49f^{high}-PE and CD24^{low}-APC sorted basal epithelial cells from *Hi-Myc:R26$^{mTmG/+}$:Gli1$^{CreER/+}$* (HiMyc) and *Hi-Myc:R26$^{mTmG/+}$:Ar$^{L/Y}$:Gli1$^{CreER/+}$* (HiMyc-ARKO) mice. **b-c** Representative Co-IF staining of CD49f^{high} sorted cells from HiMyc and HiMyc-ARKO mice. Pink arrow shows double positive for CK14 and CK8 in the indicated genotype. Scale bars, 40 µm. **d** Quantification of organoid forming efficiency showing the percentage of organoids above 50 µm diameter per total cells seeded at day 0 in a well. The center line represents the median value, the box borders represent the lower and upper quartiles (25% and 75% percentiles, respectively) and the ends of the bottom and top whiskers represent the minimum and maximum values, respectively, for six independent samples over three biological replicates. Two-sided Student's *t*-test for HiMyc versus HiMyc-ARKO, $^{**}p < 0.01$. **e-h** Representative brightfield images depicting organoid morphology and structure observed in indicated group (Vehicle); scale bars, 400 µm. Representative images of H&E staining from organoid tissue sections from each group; scale bars, 100 µm and 25 µm. Representative IHC analyses for Myc, AR, pIGF1R, β-cat, CK14, and CK8 on adjacent sections in vehicle-treated organoids from indicated genotypes. Scale bars, 25 µm. **i** Quantification for percentage of nuclear Myc^{+} epithelial cells per total epithelial cells from organoids with the indicated treatments (IGF1 100 ng/ml, IGFBP3 1 mg/ml) and genotypes. Data are represented as mean ± SD of three biological replicates. Two-sided Student's *t*-test for IGF1-treated groups versus IGF1+IGFBP3-treated groups, $^{**}p < 0.01$. **j-k** Representative H&E and IHC analyses for Myc, AR, and pIGF1R on adjacent sections in prostatic epithelial organoids derived from basal epithelia of wild type mice with indicated treatments. Scale bars, 100 µm, 25 µm. For **d** and **i**, source data and the exact *p*-values are provided in the Source Data file.

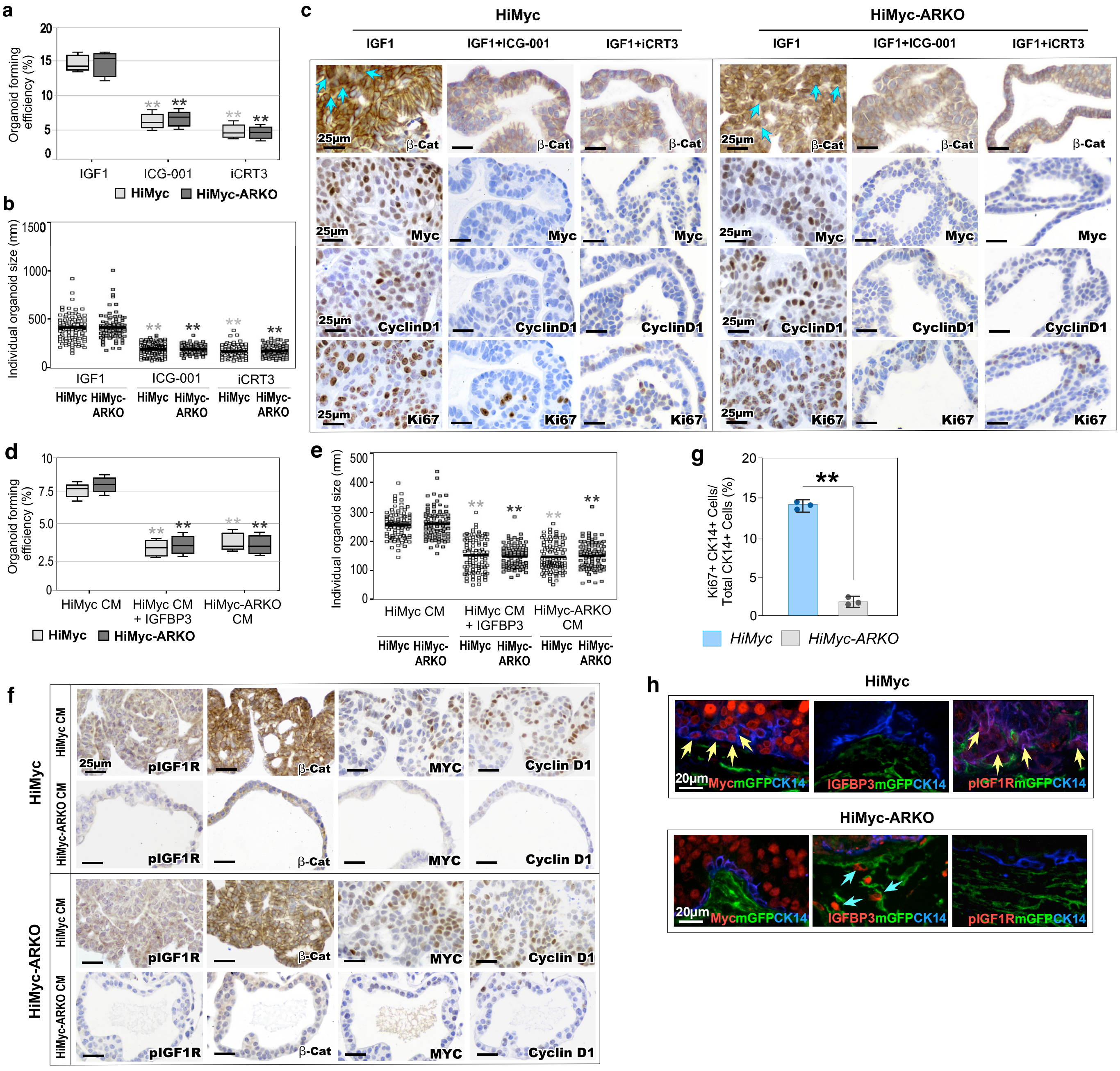

Supplementary Fig.6 Analyses of prostatic epithelial organoid growth induced by IGF and Wnt axes. a-c Organoids derived from prostatic epithelial cells from *Hi-Myc:R26*$^{mTmG/+}$*:Gli1*$^{CreER/+}$ (HiMyc) and *Hi-Myc:R26*$^{mTmG/+}$*:Ar*$^{L/Y}$*:Gli1*$^{CreER/+}$ (HiMyc-ARKO) mice treated with IGF1 (100 ng/ml), IGF1 (100 ng/ml) + ICG-001 (5 μM), or IGF1 (100 ng/ml) + iCRT3 (5 μM). Quantification of organoid forming efficiency showing the percentage of organoids derived from above 50 μm diameter per total cells seeded at day 0 in a well (**a**). Quantification of individual organoid size. Organoids (*n* = 95) per treatment group examined over two independent experiments. The center line represents the median value in each group. Two-sided Student's *t*-test, **$p < 0.01$ (**b**). Representative IHC images for the indicated antibodies in HiMyc and HiMyc-ARKO organoids with the indicated treatments (**c**). **d-f** Organoids derived from prostatic epithelial cells from HiMyc and HiMyc-ARKO mice treated with HiMyc conditioned media (CM), HiMyc CM + IGFBP3 (1 μg/ml), or HiMyc-ARKO CM**.** Quantification of organoid forming efficiency showing the percentage of organoids derived from above 50 μm diameter per total cells seeded at day 0 in a well (**d**). Quantification of individual organoid size. Organoids (*n* = 93) per treatment group examined over two independent experiments. The center line represents the median value in each group. Two-sided Student's *t*-test, **$p < 0.01$ (**e**) Representative IHC staining for the indicated antibodies in HiMyc and HiMyc-ARKO organoids with the indicated treatments (**f**). **g** Quantification for the percentage of Ki67^{+} CK14^{+} cells per total CK14^{+} cells in HiMyc and HiMyc-ARKO mice (**Fig. 7c**). Data are represented as mean ± SD of three biological replicates. Two-sided *t*-test, **$p < 0.01$. **h** Representative triple-IF staining for the indicated antibodies in HiMyc and HiMyc-ARKO mice with *Gli1*$^{CreER/+}$ activation at 2-months of age. Scale bars, 20 μm. In **a** and **d**, the center line represents the median value, the box borders represent the lower and upper quartiles (25% and 75% percentiles, respectively) and the ends of the bottom and top whiskers represent the minimum and maximum values, respectively, for six independent samples over three biological replicates. Two-sided Student's *t*-test, **$p < 0.01$. For **a**, **b**, **d**, **e**, and **g**, source data and the exact *p*-values are provided in the Source Data file.

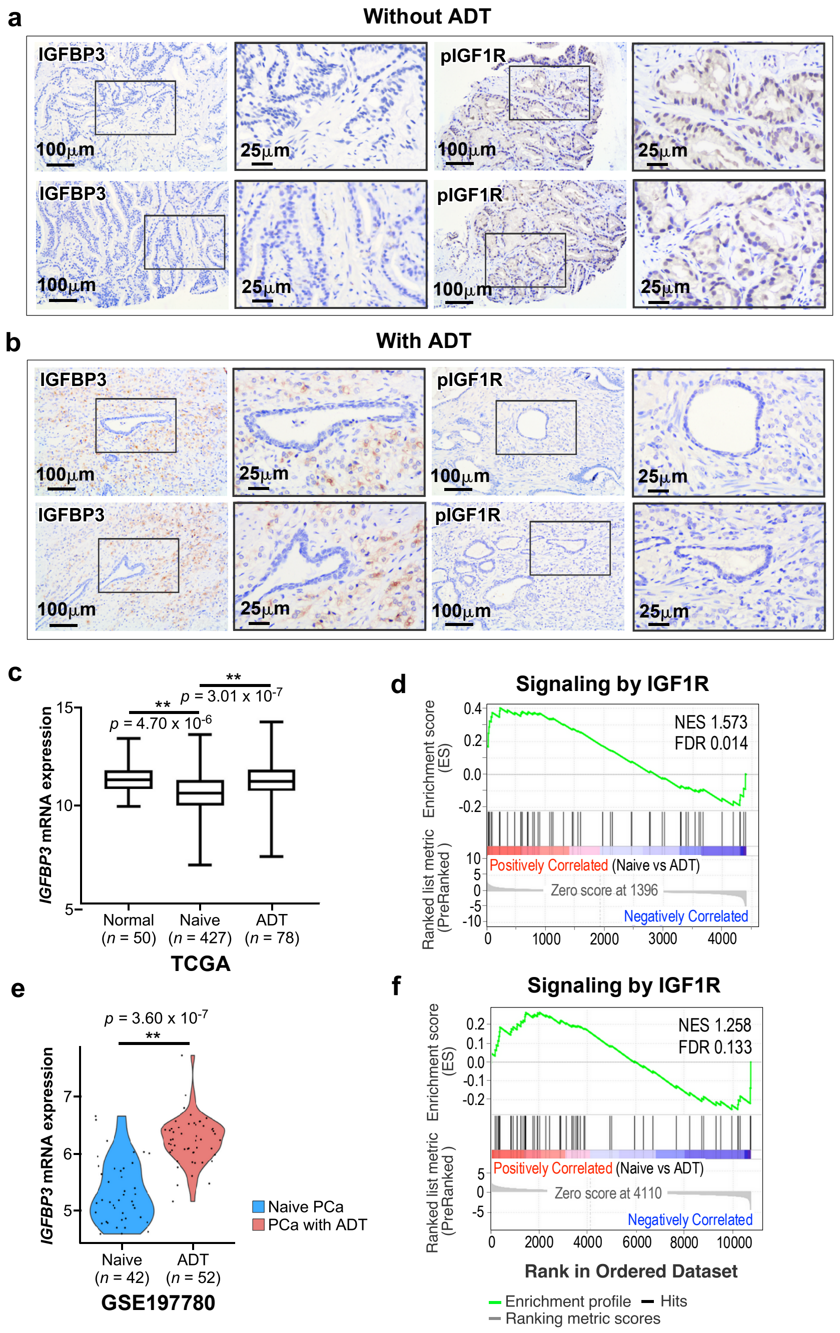

Supplementary Fig.7 Examining IGF1 signaling and IGFBP3 expression in human prostate cancer samples. a-b Representative IHC images of human prostate cancer samples with the indicated antibodies and treatments. Scale bars, 100µm and 25µm. ADT, androgen deprivation therapy. **c** *IGFBP3* mRNA expression of human prostate cancer patients with the indicated conditions and *n* number using The Cancer Genome Atlas (TCGA) database. Box and whisker plots represent minimum expression for the bottom whisker, maximum expression for the top whisker and median shown with the middle line. Statistical significance calculated by two-sided Student's *t*-test, $^{**}p < 0.01$. **d** Gene Set Enrichment Analysis (GSEA) enrichment plot depicting signaling by insulin-like growth factor 1 receptor (IGF1R) pathway significantly up-regulated in Naïve prostate carcinomas versus ADT-treated counterparts from the TCGA database. NES, normalized enrichment score; FDR, false discovery rate. See also "Methods". **e** Violin plot for *IGFBP3* mRNA expression in RNA-seq data from primary PCa before and after enzalutamide therapy (GSE197780). Two-sided Student's *t*-test, $^{**}p < 0.01$. **f**, GSEA enrichment plot depicting signaling by IGF1R pathway significantly up-regulated in Naïve prostate cancer versus ADT-treated counterparts from GSE197780. Please also see "Methods".

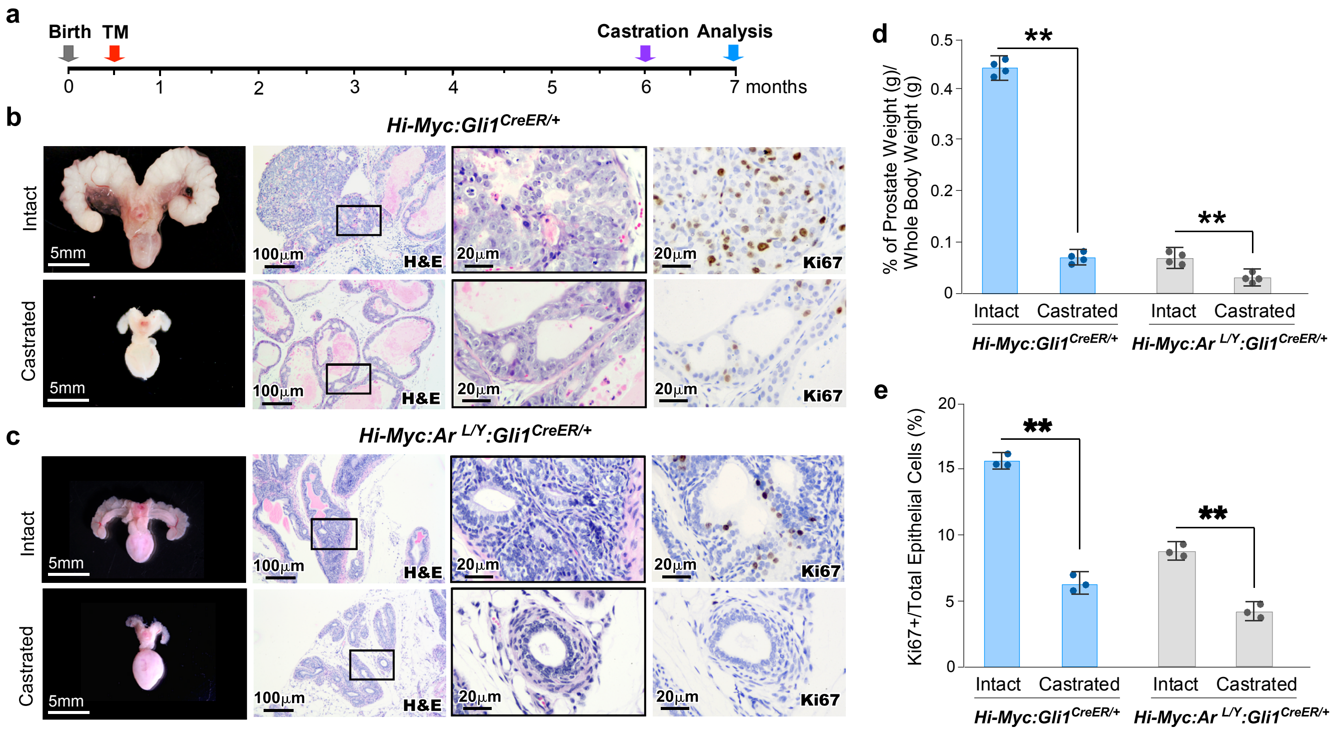

Supplementary Fig.8. Androgen deprivation impairs prostate epithelial oncogenic growth. a Schematic of experimental timeline for castration of *Hi-Myc:Gli1*$^{CreER/+}$ (HiMyc) and *Hi-Myc:Ar*$^{L/Y}$*:Gli1*$^{CreER/+}$ (HiMyc-ARKO) mice. TM, tamoxifen. **b-c** Representative gross, hematoxylin-eosin (H&E), and immunohistochemistry for Ki67 of prostate tissues from intact and castrated HiMyc and HiMyc-ARKO mice. Scale bars, 5 mm, 100 µm and 20 µm. **d** Ratio of prostate weight versus whole body weight as percentages of 7-month-old intact or castrated HiMyc and HiMyc-ARKO mice. Data are represented as mean ± SD of four biological replicates. Two-sided Student' s *t*-test, ***p* < 0.01. **e** Quantification of the percentage of Ki67^{+} cells per total epithelial cells. Data are represented as mean ± SD of three biological replicates. Two-sided Student' s *t*-test for HiMyc intact versus HiMyc castrated or HiMyc-ARKO intact versus HiMyc-ARKO castrated, ***p* < 0.01. Source data and the exact *p*-values are provided in the Source Data file.

Supplementary Table 1: List of antibodies used for IHC and IF staining in this study.

Antibody	Vendors and Cat #	Species	Working dilution
β-catenin	BD Transduction Laboratories #610154	mouse IgG	1:200
β-catenin	Santa Cruz #sc-7199	rabbit IgG	1:500
AR	ThermoFisher #PA1-9005	goat IgG	1:500
MYC	Abcam #ab168727	rabbit IgG	1:1000
MYC	NovusBio #AF3696	goat IgG	1:500
Ki67	Cell Signaling #9129	rabbit IgG	1:500
pAKT	Cell Signaling #9271	rabbit IgG	1:50
CK8	Convance #MMS-162P	mouse IgG	1:2000
CK8	Abcam #ab59400	rabbit IgG	1:200
CK14	Abcam #ab7800	mouse IgG	1:200
IGF1R	Cell Signaling #9449	rabbit IgG	1:750
pIGF1R	Bioss Antibodies #bs-5447R	rabbit IgG	1:350
Cyclin D1	Abcam #ab16663	rabbit IgG	1:200
TCF7L2	NovusBio #NBP2-67618	rabbit IgG	1:250
CD44	Santa Cruz #sc-18849	rat IgG	1:200
GFP	Cell Signaling #2956	rabbit IgG	1:200
GFP	Cell Signaling #2955	mouse IgG	1:200
GFP	Abcam #ab13970	chicken IgG	1:2000
IGFBP3	Santa Cruz #sc-365936	mouse IgG	1:500
Vimentin	BioLegend #919101	chicken IgG	1:2000
PDGFRβ	Abcam #ab32570	rabbit IgG	1:500
P63	BioLegend #W15093	mouse IgG	1:2000
P63	Gene Tex #GTX102425	rabbit IgG	1:1000
pGSK3B	Cell Signaling #9331S	rabbit IgG	1:50
pGSK3B	Proteintech #67558-1-Ig	mouse IgG	1:600
TWIST1	gifted by Carlotta Glackin (Beckman Research Institute)[1]	rabbit IgG	1:800
Biotinylated anti-mouse	Vector Laboratories #BA-9200	goat IgG	1:750
Biotinylated anti-rabbit	Vector Laboratories #BA-1000	goat IgG	1:750
Biotinylated anti-goat	Vector Laboratories #BA-5000	rabbit IgG	1:750
Biotinylated anti-rat	Vector Laboratories #BA-9400	goat IgG	1:750
Goat anti-rabbit 488	Invitrogen #A11034	goat IgG	1:500
Goat anti-mouse 488	Invitrogen #A11001	goat IgG	1:500
Goat anti-rabbit 594	Invitrogen #A11012	goat IgG	1:500
Goat anti-mouse 594	Invitrogen #A11005	goat IgG	1:500
Goat anti-chicken 647	Invitrogen #A31571	goat IgG	1:500
Donkey anti-rabbit 488	Invitrogen #A21206	donkey IgG	1:500
Donkey anti-mouse 488	Invitrogen #A21202	donkey IgG	1:500
Donkey anti-rabbit 594	Invitrogen #A21207	donkey IgG	1:500
Donkey anti-mouse 594	Invitrogen #A21203	donkey IgG	1:500
Donkey anti-goat 647	Invitorgen #A21447	donkey IgG	1:500

Supplementary Table 2: List of primers used for qRT-PCR and ChIP-qPCR in this study.

Experiment	Genes	Primer name	Sequences
qRT-PCR	*hMYC*	MYC-Forward	5’ - GTC AAG AGG CGA ACA CAC AAC - 3’
		MYC-Reverse	5’ - TTG GAC GGA CAG GAT GTA TGC - 3’
	Ctnnb1	Ctnnb1-Forward	5’ - TGA CAC CTC CCA AGT CCT TT - 3’
		Ctnnb1-Reverse	5’ - TTG CAT ACT GCC CGT CAA T - 3’
	Ccnd1	Ccnd1-Forward	5’ - GCG TAC CCT GAC ACC AAT CTC - 3’
		Ccnd1-Reverse	5’ - ACT TGA AGT AAG ATA CGG AGG GC - 3’
	Igf1r	Igf1r-Forward	5’ - TGA CAT CCG CAA CGA CTA TCA - 3’
		Igf1r_Reverse	5’ - CCA GTG CGT AGT TGT AGA AGA GT- 3
	Cd44	Cd44-Forward	5’ - TCG ATT TGA ATG TAA CCT GCC G - 3’
		Cd44-Reverse	5’ - CAG TCC GGG AGA TAC TGT AGC - 3’
	Tcf7l2	Tcf7l2-Forward	5’ - GCA TCC CTC ACC CGG CCA TC - 3’
		Tcf7l2-Reverse	5’ - GCC ACC TGC GCC CGA GAA TC - 3’
	Hras	Hras-Forward	5’ - GCT GTA GAA GCT ATG ACA GAA TAC - 3’
		Hras-Reverse	5’ - GCT GTG TCT AAG ATG TCC AGT AG - 3’
	Grb2	Grb2-Forward	5’ - CCT GGA CTT AGC ATT GTG AG - 3’
		Grb2-Reverse	5’ - TTA TCA TCA GCA GGG AGA GC - 3’
	Irs2	Irs2-Forward	5’ - CCA GTA AAC GGA GGT GGC TAC A - 3’
		Irs2-Reverse	5’ - CCA TAG ACA GCT TGG AGC CAC A - 3’
	Akt1	Akt1-Forward	5’ - GGA CTA CTT GCA CTC CGA GAA G - 3’
		Akt1-Reverse	5’ - CAT AGT GGC ACC GTC CTT GAT C - 3’
	Ar	Ar-Forward	5’- AAA CTT CTT TCG CTG GGG CTT C - 3’
		Ar-Reverse	5’- AGA ACA GAA CAC TAG CGC TTG G - 3’
	Igfbp3	Igfbp3-Forward	5’- CCA GGA AAC ATC AGT GAG TCC - 3’
		Igfbp3-Reverse	5’- GGA TGG AAC TTG GAA TCG GTC A - 3’
	Pdgfrb	Pdgfrb-Forward	5’ - AGG ACA ACC GTA CCT TGG GTG ACT - 3’
		Pdgfrb-Reverse	5’ - CAG TTC TGA CAC GTA CCG GGT CTC - 3’
	Twist1	Twist1-Forward	5’ - GAT TCA GAC CCT CAA ACT GGC G - 3’
		Twist1-Reverse	5’ - AGA CGG AGA AGG CGT AGC TGA G - 3’
	Sox9	Sox9-Forward	5’ - GCG TCA ACG GCT CCA GCA AGA - 3’
		Sox9-Reverse	5’ - GCC AGC TTG CAC GTC GGT TTT G - 3’
	Foxf1	Foxf1-Forward	5’ - CCT TCA CCA AAA CAG TCA CAA CGG - 3’
		Foxf1-Reverse	5’ - TCA CCT CAC ATC ACA CAC GGC TTG - 3’
	Il11	Il11-Forward	5’ - AAT TCC CAG CTG ACG GAG ATC ACA - 3’
		Il11-Reverse	5’ - TCT ACT CGA AGC CTT GTC AGC ACA - 3’
	Cxcl10	Cxcl10-Forward	5’ - ATC ATC CCT GCG AGC CTA TCC T - 3’
		Cxcl10-Reverse	5’ - GAC CTT TTT TGG CTA AAC GCT TTC - 3’
	Ppia	Ppia-Forward	5’ - TGT GCC AGG GTG GTG ACT TT - 3’
		Ppia-Reverse	5’ - CGT TTG TGT TTG GTC CAG CAT - 3’
ChIP-qPCR	*Igfbp3*	Igfbp3-Forward	5’ - TGC GCA CAG AGA GCA GGT G - 3’
		Igfbp3-Reverse	5’ - CTC CCG ACG AAG CGG GAA C - 3’
	Untr4	Untr4-Forward	5’ - CTC CCT CCT GTG CTT CTC AG - 3'
		Untr4-Reverse	5’ - AAT GAA CGT GTC TCC CAG AA - 3'

Supplementary Reference:

1. Elias *et al*. TWIST is expressed in human gliomas and promotes invasion. *Neoplasia* **7**(9), 824-837 (2005).
