# Supplementary material for: Stromal androgen signaling acts as tumor niches to drive prostatic basal epithelial progenitor-initiated oncogenesis
Source: Nat Commun. 2022 Nov 2;13:6552. doi: 10.1038/s41467-022-34282-w (PMC9630272; doi:10.1038/s41467-022-34282-w)
Supplement: Supplementary file 7 — Reporting Summary [file 41467_2022_34282_MOESM7_ESM.pdf]

nature portfolio

Corresponding author(s): Zijie Sun MD, PhD

Last updated by author(s): Oct 13, 2022

Reporting Summary

Nature Portfolio wishes to improve the reproducibility of the work that we publish. This form provides structure for consistency and transparency in reporting. For further information on Nature Portfolio policies, see our Editorial Policies and the Editorial Policy Checklist.

Statistics

For all statistical analyses, confirm that the following items are present in the figure legend, table legend, main text, or Methods section.

n/a	Confirmed	
☐	☒	The exact sample size (*n*) for each experimental group/condition, given as a discrete number and unit of measurement
☐	☒	A statement on whether measurements were taken from distinct samples or whether the same sample was measured repeatedly
☐	☒	The statistical test(s) used AND whether they are one- or two-sided<br>*Only common tests should be described solely by name; describe more complex techniques in the Methods section.*
☒	☐	A description of all covariates tested
☐	☒	A description of any assumptions or corrections, such as tests of normality and adjustment for multiple comparisons
☐	☒	A full description of the statistical parameters including central tendency (e.g. means) or other basic estimates (e.g. regression coefficient) AND variation (e.g. standard deviation) or associated estimates of uncertainty (e.g. confidence intervals)
☐	☒	For null hypothesis testing, the test statistic (e.g. *F*, *t*, *r*) with confidence intervals, effect sizes, degrees of freedom and *P* value noted<br>*Give P values as exact values whenever suitable.*
☒	☐	For Bayesian analysis, information on the choice of priors and Markov chain Monte Carlo settings
☒	☐	For hierarchical and complex designs, identification of the appropriate level for tests and full reporting of outcomes
☐	☒	Estimates of effect sizes (e.g. Cohen's *d*, Pearson's *r*), indicating how they were calculated

Our web collection on statistics for biologists contains articles on many of the points above.

Software and code

Policy information about availability of computer code

Data collection	Illumina Novaseq 6000 and 10× Genomics Cell Ranger pipeline (3.1.0) were used for single-cell sequencing data collection. Axio Lab A1 microscope, Canon EOS 1000D camera with AxioVision software (Carl Zeiss, AxioVision Rel. 4.8 Ink.), Nikon ECLIPSE E800 epifluorescence microscope, and QImaging RETGA EXi camera with QCapture software (QImaging v7.0) were used for imaging. RNA-seq data for prostate cancer patients were downloaded from TCGA database at UCSC Xena (Goldman, M.J. et al., Visualizing and interpreting cancer genomics data via the Xena platform. Nat Biotechnol (2020); https://doi.org/10.1038/s41587-020-0546-8). The gene expression was determined experimentally using the Illumina HiSeq 2000 RNA Sequencing platform by the University of North Carolina TCGA genome characterization center.
Data analysis	The Software R (4.2.1) was used with the Seurat package (3.2.1) for single-cell sequencing data analysis. Pathway analysis was performed using Gene Set Enrichment Analysis (GSEA 4.1.0; Subramanian A, et al. Proc Natl Acad Sci U S A 102, 15545-15550 (2005)). GraphPad Prism software v6 was used to generate graphs. ImageJ was used to analyze counting cells and organoid culture experiment images. Included in "Methods" section. R scripts used to process sequencing data are available in “GitHub repository [https://github.com/wk-kim/HiMYC-ARKO-Gli1_Stromal_AR_Prostate_Tumorigenesis]”. Included in methods section.

For manuscripts utilizing custom algorithms or software that are central to the research but not yet described in published literature, software must be made available to editors and reviewers. We strongly encourage code deposition in a community repository (e.g. GitHub). See the Nature Portfolio guidelines for submitting code & software for further information.

Data

Policy information about availability of data

All manuscripts must include a data availability statement. This statement should provide the following information, where applicable:

- Accession codes, unique identifiers, or web links for publicly available datasets
- A description of any restrictions on data availability
- For clinical datasets or third party data, please ensure that the statement adheres to our policy

Raw data of single-cell RNA sequencing have been deposited in Gene Expression Omnibus (GEO) under accession number GSE174471 [https://www.ncbi.nlm.nih.gov/geo/query/acc.cgi?acc=GSE174471]. Source data for this study are provided with this paper. All relevant data in this study are available within the article, Supplementary information, Supplementary Data, or Source Data. RNA-seq data for prostate cancer patients were downloaded from TCGA database at UCSC Xena. Drug treatment status per sample was downloaded from the cBioportal website (http://www.cbioportal.org/). The GSE197780 [https://www.ncbi.nlm.nih.gov/geo/query/acc.cgi?acc=GSE197780] dataset from the GEO database was used for validation. Statement was included in methods section.

Field-specific reporting

Please select the one below that is the best fit for your research. If you are not sure, read the appropriate sections before making your selection.

☒ Life sciences ☐ Behavioural & social sciences ☐ Ecological, evolutionary & environmental sciences

For a reference copy of the document with all sections, see nature.com/documents/nr-reporting-summary-flat.pdf

Life sciences study design

All studies must disclose on these points even when the disclosure is negative.

Sample size	Sample size estimated in this study was based on number of available, statistical methods, and previous reported data with related mouse models. Sample sizes were also based on reproducibility of the results and in which statistical significance using two-sided Student's t-test was observed and are consistent with our previous works and publications in the field (Le V, et al. Loss of androgen signaling in mesenchymal sonic hedgehog responsive cells diminishes prostate development, growth, and regeneration. PLoS Genet 16, e1008588 (2020), Kim WK, et al. Aberrant androgen action in prostatic progenitor cells induces oncogenesis and tumor development through IGF1 and Wnt axes. Nat Commun 13, 4364 (2022).) At least 3 mice per condition were used. This represents a >98% probability of detecting a significant change if alpha is set at 0.05 and SDs are 8% of average. All representative images with consistent results from at least three biological replicates are shown to show statistical significance.
Data exclusions	None of the were data excluded. All of generated male mice were included in the study due to the nature of the prostate cancer models.
Replication	All experiments were performed at least three independent times and/or with sufficient samples per group for statistical significance. All replication attempts were successful.
Randomization	All of experimental mice with different genotypes were included without selection in this study. Negative control groups such as genotype or vehicle treatment were included in all experiments. For other experiments and analysis, randomization and group allocation is not relevant or applicable in this study as all experimental mice were included from age-matched littermates and were all under the same conditions in each comparison.
Blinding	Blinding methods were used for assessing the pathological changes in prostate tumor developments for each genotype/mouse model used in this study. Investigators were blinded to the groups in the analysis for all experiments.

Behavioural & social sciences study design

All studies must disclose on these points even when the disclosure is negative.

Study description	*Briefly describe the study type including whether data are quantitative, qualitative, or mixed-methods (e.g. qualitative cross-sectional, quantitative experimental, mixed-methods case study).*
Research sample	*State the research sample (e.g. Harvard university undergraduates, villagers in rural India) and provide relevant demographic information (e.g. age, sex) and indicate whether the sample is representative. Provide a rationale for the study sample chosen. For studies involving existing datasets, please describe the dataset and source.*
Sampling strategy	*Describe the sampling procedure (e.g. random, snowball, stratified, convenience). Describe the statistical methods that were used to predetermine sample size OR if no sample-size calculation was performed, describe how sample sizes were chosen and provide a rationale for why these sample sizes are sufficient. For qualitative data, please indicate whether data saturation was considered, and what criteria were used to decide that no further sampling was needed.*
Data collection	*Provide details about the data collection procedure, including the instruments or devices used to record the data (e.g. pen and paper, computer, eye tracker, video or audio equipment) whether anyone was present besides the participant(s) and the researcher, and*

whether the researcher was blind to experimental condition and/or the study hypothesis during data collection.

Timing	*Indicate the start and stop dates of data collection. If there is a gap between collection periods, state the dates for each sample cohort.*
Data exclusions	*If no data were excluded from the analyses, state so OR if data were excluded, provide the exact number of exclusions and the rationale behind them, indicating whether exclusion criteria were pre-established.*
Non-participation	*State how many participants dropped out/declined participation and the reason(s) given OR provide response rate OR state that no participants dropped out/declined participation.*
Randomization	*If participants were not allocated into experimental groups, state so OR describe how participants were allocated to groups, and if allocation was not random, describe how covariates were controlled.*

Ecological, evolutionary & environmental sciences study design

All studies must disclose on these points even when the disclosure is negative.

Study description	*Briefly describe the study. For quantitative data include treatment factors and interactions, design structure (e.g. factorial, nested, hierarchical), nature and number of experimental units and replicates.*
Research sample	*Describe the research sample (e.g. a group of tagged Passer domesticus, all Stenocereus thurberi within Organ Pipe Cactus National Monument), and provide a rationale for the sample choice. When relevant, describe the organism taxa, source, sex, age range and any manipulations. State what population the sample is meant to represent when applicable. For studies involving existing datasets, describe the data and its source.*
Sampling strategy	*Note the sampling procedure. Describe the statistical methods that were used to predetermine sample size OR if no sample-size calculation was performed, describe how sample sizes were chosen and provide a rationale for why these sample sizes are sufficient.*
Data collection	*Describe the data collection procedure, including who recorded the data and how.*
Timing and spatial scale	*Indicate the start and stop dates of data collection, noting the frequency and periodicity of sampling and providing a rationale for these choices. If there is a gap between collection periods, state the dates for each sample cohort. Specify the spatial scale from which the data are taken*
Data exclusions	*If no data were excluded from the analyses, state so OR if data were excluded, describe the exclusions and the rationale behind them, indicating whether exclusion criteria were pre-established.*
Reproducibility	*Describe the measures taken to verify the reproducibility of experimental findings. For each experiment, note whether any attempts to repeat the experiment failed OR state that all attempts to repeat the experiment were successful.*
Randomization	*Describe how samples/organisms/participants were allocated into groups. If allocation was not random, describe how covariates were controlled. If this is not relevant to your study, explain why.*
Blinding	*Describe the extent of blinding used during data acquisition and analysis. If blinding was not possible, describe why OR explain why blinding was not relevant to your study.*

Did the study involve field work? ☐ Yes ☐ No

Field work, collection and transport

Field conditions	*Describe the study conditions for field work, providing relevant parameters (e.g. temperature, rainfall).*
Location	*State the location of the sampling or experiment, providing relevant parameters (e.g. latitude and longitude, elevation, water depth).*
Access & import/export	*Describe the efforts you have made to access habitats and to collect and import/export your samples in a responsible manner and in compliance with local, national and international laws, noting any permits that were obtained (give the name of the issuing authority, the date of issue, and any identifying information).*
Disturbance	*Describe any disturbance caused by the study and how it was minimized.*

Reporting for specific materials, systems and methods

We require information from authors about some types of materials, experimental systems and methods used in many studies. Here, indicate whether each material, system or method listed is relevant to your study. If you are not sure if a list item applies to your research, read the appropriate section before selecting a response.

Materials & experimental systems

n/a	Involved in the study
☐	☒ Antibodies
☒	☐ Eukaryotic cell lines
☒	☐ Palaeontology and archaeology
☐	☒ Animals and other organisms
☐	☒ Human research participants
☒	☐ Clinical data
☒	☐ Dual use research of concern

Methods

n/a	Involved in the study
☒	☐ ChIP-seq
☒	☐ Flow cytometry
☒	☐ MRI-based neuroimaging

Antibodies

Antibodies used	Included in Supplementary Table 1 and referred in methods section. All antibodies for immunohistochemistry and immonfluorescence used were from commercial sources: β-catenin BD Transduction Laboratories #610154 mouse IgG 1:200, β-catenin Santa Cruz #sc-7199 rabbit IgG 1:500, AR ThermoFisher #PA1-9005 goat IgG 1:500, MYC Abcam #ab168727 rabbit IgG 1:1000, MYC NovusBio #AF3696 goat IgG 1:500, Ki67 Cell Signaling #9129 rabbit IgG 1:500, pAKT Cell Signaling #9271 rabbit IgG 1:50, CK8 Convance #MMS-162P mouse IgG 1:2000, CK14 Abcam #ab7800 mouse IgG 1:200, IGF1R Cell Signaling #9750 rabbit IgG 1:750, pIGF1R Bioss Antibodies #bs-5447R rabbit IgG 1:350, Cyclin D1 Abcam #ab16663 rabbit IgG 1:200, TCF7L2 NovusBio #NBP2-67618 rabbit IgG 1:250, CD44 Santa Cruz #sc-18849 rat IgG 1:200, GFP Cell Signaling #2956 rabbit IgG 1:200, GFP Cell Signaling #2955 mouse IgG 1:200, GFP Abcam #ab13970 chicken IgG 1:2000, IGFBP3 Santa Cruz #sc-365936 mouse IgG 1:500, Vimentin BioLegend #919101 chicken IgG 1:2000, PDGFRβ Abcam #ab32570 rabbit IgG 1:500, Biotinylated anti-mouse Vector Laboratories #BA-9200 goat IgG 1:750, Biotinylated anti-rabbit Vector Laboratories #BA-1000 goat IgG 1:750, Biotinylated anti-goat Vector Laboratories #BA-5000 rabbit IgG 1:750, Biotinylated anti-rat Vector Laboratories #BA-9400 goat IgG 1:750, Goat anti-rabbit 488 Invitrogen #A11034 goat IgG 1:500, Goat anti-mouse 488 Invitrogen #A11001 goat IgG 1:500, Goat anti-rabbit 594 Invitrogen #A11012 goat IgG 1:500, Goat anti-mouse 594 Invitrogen #A11005 goat IgG 1:500, Goat anti-chicken 647 Invitrogen #A21449 goat IgG 1:500, Donkey anti-rabbit 488 Invitrogen #A21206 donkey IgG 1:500, Donkey anti-mouse 488 Invitrogen #A21202 donkey IgG 1:500, Donkey anti-rabbit 594 Invitrogen #A21207 donkey IgG 1:500, Donkey anti-mouse 594 Invitrogen #A21203 donkey IgG 1:500, Donkey anti-goat 647 Invitrogen #A21447 donkey IgG 1:500. CK8 Abcam #ab59400 rabbit IgG 1:200. P63 BioLegend #W15093 mouse IgG 1:2000. P63 Gene Tex #GTX102425 rabbit IgG 1:1000. pGSK3B Cell Signaling #9331S rabbit IgG 1:50. pGSK3B Proteintech #67558-1-Ig mouse IgG 1:600. TWIST1 antibody was kindly gifted by Dr. Carlotta Glackin from Beckman Research Institute, Duarte, CA.
Validation	All antibodies used were from commercial sources and vendor confirmed species reactivity and references listed on vendor website. https://www.bdbiosciences.com/en-us/products/reagents/microscopy-imaging-reagents/immunofluorescence-reagents/purified-mouse-anti-catenin.610154, https://www.scbt.com/p/beta-catenin-antibody-h-102?requestFrom=search, https://www.thermofisher.com/antibody/product/Androgen-Receptor-Antibody-Polyclonal/PA1-9005, https://www.abcam.com/c-myc-antibody-y69-bsa-and-azide-free-ab168727.html, https://www.novusbio.com/products/c-myc-antibody_af3696, https://www.cellsignal.com/products/primary-antibodies/ki-67-d3b5-rabbit-mab/9129, https://www.cellsignal.com/products/primary-antibodies/phospho-akt-ser473-antibody/9271, https://www.biolegend.com/en-us/products/cytokeratin-8-monoclonal-antibody-purified-10929?GroupID=GROUP26, https://www.abcam.com/Cytokeratin-14-antibody-LL002-ab7800.html, https://www.cellsignal.com/products/primary-antibodies/igf-i-receptor-b-d23h3-xp-rabbit-mab/9750, https://www.biossusa.com/products/bs-5447r, https://www.abcam.com/cyclin-d1-antibody-sp4-ab16663.html, https://www.novusbio.com/products/tcf7l2-antibody-sc06-90_nbp2-67618, https://www.scbt.com/p/hcam-antibody-im7/, https://www.cellsignal.com/products/primary-antibodies/gfp-d5-1-xp-rabbit-mab/2956, https://www.cellsignal.com/products/primary-antibodies/gfp-4b10-mouse-mab/2955, https://www.abcam.com/GFP-antibody-ab13970.html, https://www.scbt.com/p/igfbp3-antibody-b-5, https://www.biolegend.com/en-us/products/purified-anti-vimentin-antibody-11598?GroupID=GROUP26, https://www.abcam.com/pdgfr-alpha--pdgfr-beta-antibody-y92-c-terminal-ab32570.html, https://vectorlabs.com/biotinylated-goat-anti-mouse-igg-antibody.html, https://vectorlabs.com/biotinylated-goat-anti-rabbit-igg-antibody.html, https://vectorlabs.com/biotinylated-rabbit-anti-goat-igg-antibody.html, https://vectorlabs.com/biotinylated-goat-anti-rat-igg-antibody.html, https://www.thermofisher.com/antibody/product/Goat-anti-Rabbit-IgG-H-L-Cross-Adsorbed-Secondary-Antibody-Polyclonal/A-11012, https://www.thermofisher.com/antibody/product/Goat-anti-Mouse-IgG-H-L-Cross-Adsorbed-Secondary-Antibody-Polyclonal/A-11005, https://www.thermofisher.com/antibody/product/Goat-anti-Chicken-IgY-H-L-Secondary-Antibody-Polyclonal/A-21449, https://www.thermofisher.com/antibody/product/Donkey-anti-Rabbit-IgG-H-L-Highly-Cross-Adsorbed-Secondary-Antibody-Polyclonal/A-21206, https://www.thermofisher.com/antibody/product/Donkey-anti-Mouse-IgG-H-L-Highly-Cross-Adsorbed-Secondary-Antibody-Polyclonal/A-21202, https://www.thermofisher.com/antibody/product/Donkey-anti-Rabbit-IgG-H-L-Highly-Cross-Adsorbed-Secondary-Antibody-Polyclonal/A-21207, https://www.thermofisher.com/antibody/product/Donkey-anti-Mouse-IgG-H-L-Highly-Cross-Adsorbed-Secondary-Antibody-Polyclonal/A-21203, https://www.thermofisher.com/antibody/product/Donkey-anti-Goat-IgG-H-L-Cross-Adsorbed-Secondary-Antibody-Polyclonal/A-21447. ab59400, https://www.abcam.com/Cytokeratin-8-antibody-ab59400.html. W15093, https://www.biolegend.com/en-us/products/purified-anti-tp63-antibody-13251?GroupID=GROUP26. GTX102425, https://www.genetex.com/Product/Detail/p63-antibody-N2C1-Internal/GTX102425. 9331S, https://www.cellsignal.com/products/primary-antibodies/phospho-gsk-3a-b-ser21-9-antibody/9331?site-search-type=Products&N=4294956287&Ntt=9331s&fromPage=plp&_requestid=814953. 67558-1-Ig, https://www.ptglab.com/products/GSK3B-phospho-S9-Antibody-67558-1-Ig.htm. TWIST1 Antibody: https://www.ncbi.nlm.nih.gov/pmc/articles/PMC1501937/. Antibodies were further validated by our own IHC and IF prior to experiments.

Eukaryotic cell lines

Policy information about cell lines

Cell line source(s)	*State the source of each cell line used.*
Authentication	*Describe the authentication procedures for each cell line used OR declare that none of the cell lines used were authenticated.*
Mycoplasma contamination	*Confirm that all cell lines tested negative for mycoplasma contamination OR describe the results of the testing for mycoplasma contamination OR declare that the cell lines were not tested for mycoplasma contamination.*
Commonly misidentified lines (See ICLAC register)	*Name any commonly misidentified cell lines used in the study and provide a rationale for their use.*

Palaeontology and Archaeology

Specimen provenance	*Provide provenance information for specimens and describe permits that were obtained for the work (including the name of the issuing authority, the date of issue, and any identifying information). Permits should encompass collection and, where applicable, export.*
Specimen deposition	*Indicate where the specimens have been deposited to permit free access by other researchers.*
Dating methods	*If new dates are provided, describe how they were obtained (e.g. collection, storage, sample pretreatment and measurement), where they were obtained (i.e. lab name), the calibration program and the protocol for quality assurance OR state that no new dates are provided.*

☐ Tick this box to confirm that the raw and calibrated dates are available in the paper or in Supplementary Information.

Ethics oversight	*Identify the organization(s) that approved or provided guidance on the study protocol, OR state that no ethical approval or guidance was required and explain why not.*

Note that full information on the approval of the study protocol must also be provided in the manuscript.

Animals and other organisms

Policy information about studies involving animals; ARRIVE guidelines recommended for reporting animal research

Laboratory animals	All information for laboratory animals used are included in the methods section. Mice housing conditions are under a 12 h light/dark cycle at 20-24 °C and 30-70% humidity at City of Hope Parvin Animal Facility. They are housed in ventilated cage racks with free access to food and water. All mice used in this study were from a C57BL/6 background. Mice containing the conditional Ctnnb1 allele (Ctnnb1L(ex3)) were kindly gifted from Dr. Makoto M. Taketo. PtenL/L mice were kindly provided by Dr. Hong Wu, respectively. PB-Cre4 (PBCre) mice were obtained from the NCI mouse repository (strain #: 01XF5). Gli1CreER/+ and ROSAmTmG (R26mTmG/+) reporter mice were obtained from Jackson Laboratories (stocks 18867 and 7676). ArLox/Y (ArL/Y) and Hi-Myc transgenic (Hi-Myc) mice were kindly provided by Dr. Guido Verhoeven and Dr. Charles Sawyers, respectively. Ctnnb1L(ex3)/+:PBCre were generated by intercrossing Ctnnb1L(ex3)/L(ex3) females with PBCre male mice. PtenL/+:PBCre mice were first generated by intercrossing PtenL/L females with PBCre males and then used to generate PtenL/L:PBCre. ArL/X female mice were mated with Gli1CreER/+ male mice to generate Gli1CreER/+ and ArL/Y:Gli1CreER/+ littermates. Experimental mice were generated by intercrossing ArL/X:R26mTmG/+:Gli1CreER/+ females with Hi-Myc males. Similar mating procedures were used to generate Hi-Myc:R26mTmG/+:Gli1CreER/+ and Hi-Myc:R26mTmG/+:ArL/Y:Gli1CreER/+ male mice 8 weeks old, 12 weeks old, and 24 weeks old. PBCre male mice mated with Ctnnb1L(ex3)/L(ex3) female mice and PtenL/+:PBCre male mice mated with PtenL/L female mice to generate urogenital sinus epithelium (UGE) E16.5 tissues. Gli1CreER/+ male mice were mated with ArL/X female mice for timed pregnancy to generate urogenital sinus mesenchyme (UGM) E16.5 tissues.<br>8-week-old male NOD.CB17-Prkdc(SCID)/_(Jax_001303) mice were used for tissue recombination grafting experiments.
Wild animals	This study did not involve wild animals.
Field-collected samples	This study did not involve samples collected from the field.
Ethics oversight	All experimental procedures and care of animals in this study were carried out according to the Institutional Animal Care and Use Committee (IACUC) at Beckman Research Institute at City of Hope (California, US), and approved by the IACUC. Euthanasia was performed by CO2 inhalation followed by cervical dislocation.

Note that full information on the approval of the study protocol must also be provided in the manuscript.

Human research participants

Policy information about studies involving human research participants

Population characteristics	Specimens of primary prostate cancer used in this study were isolated from either patients without hormonal treatment or patients who received Lupron treatment for 3 months in reducing the tumor volumes to achieve tumor-negative surgical margins. Human prostate cancer tissue samples were collected previously without identifiable personal and private information.

Recruitment	Informed consent was obtained from all patients. Also, patients were asked if they would be willing to donate samples. Biases may include the willingness of patients to donate samples, which likely resulted in minimal impact on this study.
Ethics oversight	The study was approved by the Institutional Review Board (IRB)-approved protocol (IRB # HS-16-00817) at the University of Southern California.

Note that full information on the approval of the study protocol must also be provided in the manuscript.

Clinical data

Policy information about clinical studies
All manuscripts should comply with the ICMJE guidelines for publication of clinical research and a completed CONSORT checklist must be included with all submissions.

Clinical trial registration	*Provide the trial registration number from ClinicalTrials.gov or an equivalent agency.*
Study protocol	*Note where the full trial protocol can be accessed OR if not available, explain why.*
Data collection	*Describe the settings and locales of data collection, noting the time periods of recruitment and data collection.*
Outcomes	*Describe how you pre-defined primary and secondary outcome measures and how you assessed these measures.*

Dual use research of concern

Policy information about dual use research of concern

Hazards

Could the accidental, deliberate or reckless misuse of agents or technologies generated in the work, or the application of information presented in the manuscript, pose a threat to:

No	Yes	
☐	☐	Public health
☐	☐	National security
☐	☐	Crops and/or livestock
☐	☐	Ecosystems
☐	☐	Any other significant area

Experiments of concern

Does the work involve any of these experiments of concern:

No	Yes	
☐	☐	Demonstrate how to render a vaccine ineffective
☐	☐	Confer resistance to therapeutically useful antibiotics or antiviral agents
☐	☐	Enhance the virulence of a pathogen or render a nonpathogen virulent
☐	☐	Increase transmissibility of a pathogen
☐	☐	Alter the host range of a pathogen
☐	☐	Enable evasion of diagnostic/detection modalities
☐	☐	Enable the weaponization of a biological agent or toxin
☐	☐	Any other potentially harmful combination of experiments and agents

ChIP-seq

Data deposition

☐ Confirm that both raw and final processed data have been deposited in a public database such as GEO.

☐ Confirm that you have deposited or provided access to graph files (e.g. BED files) for the called peaks.

Data access links<br>*May remain private before publication.*	*For "Initial submission" or "Revised version" documents, provide reviewer access links. For your "Final submission" document, provide a link to the deposited data.*
Files in database submission	*Provide a list of all files available in the database submission.*
Genome browser session<br>(e.g. UCSC)	

Provide a link to an anonymized genome browser session for "Initial submission" and "Revised version" documents only, to enable peer review. Write "no longer applicable" for "Final submission" documents.

Methodology

Replicates	Describe the experimental replicates, specifying number, type and replicate agreement.
Sequencing depth	Describe the sequencing depth for each experiment, providing the total number of reads, uniquely mapped reads, length of reads and whether they were paired- or single-end.
Antibodies	Describe the antibodies used for the ChIP-seq experiments; as applicable, provide supplier name, catalog number, clone name, and lot number.
Peak calling parameters	Specify the command line program and parameters used for read mapping and peak calling, including the ChIP, control and index files used.
Data quality	Describe the methods used to ensure data quality in full detail, including how many peaks are at FDR 5% and above 5-fold enrichment.
Software	Describe the software used to collect and analyze the ChIP-seq data. For custom code that has been deposited into a community repository, provide accession details.

Flow Cytometry

Plots

Confirm that:

☐ The axis labels state the marker and fluorochrome used (e.g. CD4-FITC).

☐ The axis scales are clearly visible. Include numbers along axes only for bottom left plot of group (a 'group' is an analysis of identical markers).

☐ All plots are contour plots with outliers or pseudocolor plots.

☐ A numerical value for number of cells or percentage (with statistics) is provided.

Methodology

Sample preparation	Describe the sample preparation, detailing the biological source of the cells and any tissue processing steps used.
Instrument	Identify the instrument used for data collection, specifying make and model number.
Software	Describe the software used to collect and analyze the flow cytometry data. For custom code that has been deposited into a community repository, provide accession details.
Cell population abundance	Describe the abundance of the relevant cell populations within post-sort fractions, providing details on the purity of the samples and how it was determined.
Gating strategy	Describe the gating strategy used for all relevant experiments, specifying the preliminary FSC/SSC gates of the starting cell population, indicating where boundaries between "positive" and "negative" staining cell populations are defined.

☐ Tick this box to confirm that a figure exemplifying the gating strategy is provided in the Supplementary Information.

Magnetic resonance imaging

Experimental design

Design type	Indicate task or resting state; event-related or block design.
Design specifications	Specify the number of blocks, trials or experimental units per session and/or subject, and specify the length of each trial or block (if trials are blocked) and interval between trials.
Behavioral performance measures	State number and/or type of variables recorded (e.g. correct button press, response time) and what statistics were used to establish that the subjects were performing the task as expected (e.g. mean, range, and/or standard deviation across subjects).

Acquisition

Imaging type(s)	*Specify: functional, structural, diffusion, perfusion.*
Field strength	*Specify in Tesla*
Sequence & imaging parameters	*Specify the pulse sequence type (gradient echo, spin echo, etc.), imaging type (EPI, spiral, etc.), field of view, matrix size, slice thickness, orientation and TE/TR/flip angle.*
Area of acquisition	*State whether a whole brain scan was used OR define the area of acquisition, describing how the region was determined.*

Diffusion MRI ☐ Used ☐ Not used

Preprocessing

Preprocessing software	*Provide detail on software version and revision number and on specific parameters (model/functions, brain extraction, segmentation, smoothing kernel size, etc.).*
Normalization	*If data were normalized/standardized, describe the approach(es): specify linear or non-linear and define image types used for transformation OR indicate that data were not normalized and explain rationale for lack of normalization.*
Normalization template	*Describe the template used for normalization/transformation, specifying subject space or group standardized space (e.g. original Talairach, MNI305, ICBM152) OR indicate that the data were not normalized.*
Noise and artifact removal	*Describe your procedure(s) for artifact and structured noise removal, specifying motion parameters, tissue signals and physiological signals (heart rate, respiration).*
Volume censoring	*Define your software and/or method and criteria for volume censoring, and state the extent of such censoring.*

Statistical modeling & inference

Model type and settings	*Specify type (mass univariate, multivariate, RSA, predictive, etc.) and describe essential details of the model at the first and second levels (e.g. fixed, random or mixed effects; drift or auto-correlation).*
Effect(s) tested	*Define precise effect in terms of the task or stimulus conditions instead of psychological concepts and indicate whether ANOVA or factorial designs were used.*

Specify type of analysis: ☐ Whole brain ☐ ROI-based ☐ Both

Statistic type for inference (See Eklund et al. 2016)	*Specify voxel-wise or cluster-wise and report all relevant parameters for cluster-wise methods.*
Correction	*Describe the type of correction and how it is obtained for multiple comparisons (e.g. FWE, FDR, permutation or Monte Carlo).*

Models & analysis

n/a	Involved in the study
☐	☐ Functional and/or effective connectivity
☐	☐ Graph analysis
☐	☐ Multivariate modeling or predictive analysis

Functional and/or effective connectivity	*Report the measures of dependence used and the model details (e.g. Pearson correlation, partial correlation, mutual information).*
Graph analysis	*Report the dependent variable and connectivity measure, specifying weighted graph or binarized graph, subject- or group-level, and the global and/or node summaries used (e.g. clustering coefficient, efficiency, etc.).*
Multivariate modeling and predictive analysis	*Specify independent variables, features extraction and dimension reduction, model, training and evaluation metrics.*
