# Supplementary material for: Stromal androgen signaling acts as tumor niches to drive prostatic basal epithelial progenitor-initiated oncogenesis
Source: Nat Commun. 2022 Nov 2;13:6552. doi: 10.1038/s41467-022-34282-w (PMC9630272; doi:10.1038/s41467-022-34282-w)
Supplement: Supplementary file 8 — Description of Additional Supplementary Files [file 41467_2022_34282_MOESM8_ESM.pdf]

Description of Additional Supplementary Files

File Name: Supplementary Data 1
Description: The list of differentially expressed genes between *hMycTg*-positive basal epithelial cells in HiMYC and those cells in HiMYC-ARKO, related to Figure 4

File Name: Supplementary Data 2
Description: The list of differentially expressed genes between *mGFP*-positive fibroblast cells in HiMYC and those cells in HiMYC-ARKO, related to Figure 5

File Name: Supplementary Data 3
Description: The list of differentially expressed genes between *mGFP*-positive and *Ar*-positive fibroblast 1 in HiMYC and *mGFP*-positive and Ar-negative fibroblast 1 in HiMYC-ARKO, related to Figure 5

File Name: Supplementary Data 4
Description: The list of differentially expressed genes between *hMycTg*-positive basal cells and *hMycTg*-positive luminal cells in HiMYC or HiMYCARKO, related to Figure 7
